# Evaluation of the rapid diagnostic test CareStart pLDH Malaria (Pf-pLDH/pan-pLDH) for the diagnosis of malaria in a reference setting

**DOI:** 10.1186/1475-2875-11-204

**Published:** 2012-06-18

**Authors:** Marloes Heutmekers, Philippe Gillet, Jessica Maltha, Annelies Scheirlinck, Lieselotte Cnops, Emmanuel Bottieau, Marjan Van Esbroeck, Jan Jacobs

**Affiliations:** 1 Faculty of Health, Medicine and Life Sciences (FHML), Maastricht University, Maastricht, The Netherlands; 2 Department of Clinical Sciences, Institute of Tropical Medicine, Antwerp, Belgium

**Keywords:** Malaria, *Plasmodium*, Diagnostic, Evaluation, RDT, Rapid diagnostic tests, pLDH, Lactate dehydrogenase, CareStart

## Abstract

**Background:**

The present study evaluated CareStart pLDH Malaria, a three-band rapid diagnostic test detecting *Plasmodium falciparum-*specific parasite lactate dehydrogenase (Pf-pLDH) and pan *Plasmodium*-specific pLDH (pan-pLDH) in a reference setting.

**Methods:**

CareStart pLDH was retrospectively and prospectively assessed with a panel of stored (n = 498) and fresh (n = 77) blood samples obtained in international travelers suspected of malaria. Both panels comprised all four *Plasmodium* species; the retrospective panel comprised also *Plasmodium* negative samples. The reference method was microscopy corrected by PCR. The prospective panel was run side-to-side with OptiMAL (Pf-pLDH/pan-pLDH) and SDFK60 (histidine-rich protein-2 (HRP-2)/pan-pLDH).

**Results:**

In the retrospective evaluation, overall sensitivity for *P. falciparum* samples (n = 247) was 94.7%, reaching 98.7% for parasite densities > 1,000/μl. Most false negative results occurred among samples with pure gametocytaemia (2/12, 16.7%) and at parasite densities ≤ 100/μl (7/12, 58.3%). None of the *Plasmodium* negative samples (n = 96) showed visible test lines. Sensitivities for *Plasmodium vivax* (n = 70), *Plasmodium ovale* (n = 69) and *Plasmodium malariae* (n = 16) were 74.3%, 31.9% and 25.0% respectively. Wrong species identification occurred in 10 (2.5%) samples and was mainly due to *P. vivax* samples reacting with the Pf-pLDH test line. Overall, Pf-pLDH test lines showed higher line intensities compared to the pan-pLDH lines (67.9% and 23.0% medium and strong line intensities for *P. falciparum*). In the prospective panel (77 *Plasmodium*-positive samples), CareStart pLDH showed higher sensitivities for *P. falciparum* compared to OptiMAL (p = 0.008), lower sensitivities for *P. falciparum* as compare to SDFK60 (although not reaching statistical significance, p = 0.08) and higher sensitivities for *P. ovale c*ompared to both OptiMAL (p = 0.03) and SDFK60 (p = 0.01). Inter-observer and test reproducibility were good to excellent.

**Conclusion:**

CareStart pLDH performed excellent for the detection of *P. falciparum,* well for *P. vivax*, but poor for *P. ovale* and *P. malariae.*

## Background

In 2009, 225 million cases of malaria occurred with 781,000 deaths, mostly due to *Plasmodium falciparum* among children in Africa [1]. In addition, yearly an estimated 30,000 international travellers fall ill with malaria after returning from malaria-endemic regions [2]. Early diagnosis and treatment are necessary to prevent severe malaria and death. Microscopy is the cornerstone for the diagnosis but requires training and expertise. Malaria rapid diagnostic tests (RDTs) are an additional value in the laboratory work-up, both in endemic settings and in the setting of travel medicine [3,4].

RDTs consist of nitrocellulose strips mostly embedded in plastic cassettes. When blood and buffer are applied, the red blood cells are lysed and the targeted antigen binds to the detecting mouse antibody which is conjugated to colloidal gold. This complex moves further along the nitrocellulose strip until the antigen binds (by another motif) to the capture antibody embedded as a transverse line on the nitrocellulose strip. As a result, the colloidal gold is concentrated on a small surface and becomes visible as a purple-red line. The non-bound conjugated antibodies move further along the strip until they are captured by goat anti-mouse antibodies, thereby generating the control line. Two-band RDTs consist of a control line and a *P. falciparum* specific test line which targets either histidine-rich protein-2 (HRP-2) or *P. falciparum* specific lactate dehydrogenase (Pf-pLDH). Three-band RDTs display three lines: a control line, a *P. falciparum*-specific line (detection of HRP-2 or Pf-pLDH) a third line detecting *P. vivax* (by a *P. vivax*- specific pLDH, Pv-pLDH) or an antigen common to all four species, either aldolase or pan-*Plasmodium*-specific pLDH (pan-pLDH).

The present study describes the diagnostic evaluation of CareStart pLDH Malaria G0121 (AccessBio Inc., Monmouth, USA, further referred to as CareStart pLDH), a three-band RDT targeting Pf-pLDH and pan-pLDH) in a reference setting.

## Methods

### Study design

CareStart pLDH was evaluated in a non-endemic reference laboratory on clinical samples obtained in international travelers suspected of malaria. The evaluation consisted of two parts: a retrospective study on a panel of stored whole blood samples and a prospective study on fresh whole blood samples. The prospective samples were run side to side with two other RDTs used as part of standard laboratory work-up of malaria-suspected samples. The reference method was microscopy corrected by polymerase chain reaction (PCR) for *Plasmodium* detection and species identification. Parasite densities were determined by microscopy. The study design was in compliance with the STARD guidelines for presentation of diagnostic studies [5].

### Patients and materials

The panel was selected from a collection of EDTA anti-coagulated blood samples which were either obtained in patients suspected of malaria presenting at the outpatient clinic of the Institute of Tropical Medicine (ITM, Antwerp, Belgium) or submitted by other Belgian laboratories for confirmation in the scope of the national reference laboratory for *Plasmodium*. The samples were obtained in international travellers and natives of endemic regions returning from visiting friends and relatives.

The retrospective panel had been obtained between February 1996 and May 2011. Samples collected at ITM were kept at room temperature (< 25°C) for a maximum of 8 hours before analysis and subsequent storage at −70°C. Samples submitted from other Belgium laboratories had been exposed to ambient temperatures for the period of shipment which was generally less than 24 hours with a maximum of 48 hours. The selected panel comprised the four *Plasmodium* species at different parasite densities, as well as *Plasmodium* negative samples. The latter were obtained in patients suspected of malaria, but negative for *Plasmodium* by microscopy, PCR and RTDs used in the standard diagnostic work-up. Samples with pure gametocytaemia were included among the *P. falciparum* species. Mixed infections were not considered. The prospective panel included fresh first samples of all patients diagnosed with malaria by microscopy between January 2011 and July 2011. Again, mixed infections were not considered.

### Reference method

Malaria diagnostics at ITM are accredited to the requirements of NBN EN ISO 15189:2007. An expert microscopist assessed all samples for the presence of *Plasmodium* parasites, species identification and parasite density according the World Health Organization (WHO) standards for microscopy with exception of the Giemsa staining that was done with pH 8.0 instead of pH 7.2 [6,7]. Thick and thin blood films were prepared and examined by light microscopy. A minimum of 200 fields was examined before a blood film was reported negative. The parasite density was obtained by counting the asexual parasites against 200 white blood cells (WBC) in thick blood films and using the WBC count or, when not available, the standard 8,000 WBC/μl, for the conversion to parasites/μl [6,7]. Four-primer real-time PCR was performed on all samples [8]. The result of microscopy corrected by PCR was considered as the reference.

### Test platforms

In the prospective study, CareStart pLDH was run side-to-side with two other RDTs used in routine diagnosis. OptiMAL pLDH (Pan, Pf) (Biorad, Marnes-la-Coquette, France), further referred to as OptiMAL, is a three-band RDT targeting Pf-pLDH and pan-pLDH. SD Bioline Ag Pf/Pan 05FK60 (Standard Diagnostics, Hagal-Dong, Korea), further referred to as SDFK60, is a three-band RDT targeting HRP-2 and pan-pLDH. All RDTs had been stored between 18°C and 24°C. In case of an absent control line the test was considered invalid and the sample was retested. When the test lines were impossible to read due to poor background clearing, the test was scored ‘unreadable’ and repeat testing was performed. For CareStart pLDH, the interpretation of the appearance of one or both test lines in the presence of a control line is as follows (Table 1–2): the presence of a unique Pf-pLDH line indicates an infection with *P. falciparum*, whereas a unique pan-pLDH test line points to an infection with one or more of the non-*falciparum* species. The presence of both a *P. falciparum* specific and pan-*Plasmodium* test line indicates an infection with *P. falciparum* or a mixed infection with *P. falciparum* and one or more of the non*-falciparum* species. A species mismatch occurred when a wrong species was identification.

**Table 1:** **Interpretation of test results for*P. falciparum***

	**Species identification by microscopy corrected by PCR**	
		**Non-*falciparum***
	***P. falciparum***	***(P.vivax, P.ovale, P.malariae)* or no parasites detected**
Only Pf-pLDH		False positive
or	True positive	/
both Pf-pLDH and pan-pLDH		species mismatch**
No test line visible	False negative	
or	/	True Negative
only pan-pLDH	species mismatch*	

* *P. falciparum* diagnosed as non-*falciparum* species.

** Non-*falciparum* species diagnosed as *P. falciparum* or as a mixed infection with *P. falciparum*.

**Table 2:** **Interpretation of test results for the non-*falciparum*species**

	**Species identification by microscopy corrected by PCR**	
	**Non-*falciparum (P.vivax, P.ovale, P.malariae)***	***P. falciparum* or no parasites detected**
		False positive
Only pan-pLDH	True positive	/
		species mismatch*
No test line visible	False negative	
or	/	True Negative
only Pf-pLDH or both Pf-pLDH and pan-pLDH	species mismatch**	

* *P. falciparum* diagnosed as non-*falciparum* species.

** Non-*falciparum* species diagnosed as *P. falciparum* or as a mixed infection with *P. falciparum*.

For the evaluation of CareStart pLDH, kits from two different lots were used. In the retrospective evaluation lot numbers AI0IL (n = 350) and DIIML (n = 148) were used, which expired in December 2011 and March 2012 respectively. In the prospective evaluation lot AI0IL (n = 98) was used.

### Test procedures

Tests were carried out in time controlled batches. They were performed in compliance with the instructions of the manufacturers, except that the transfer devices included in the kits were replaced by a transfer pipette (Finnpipette, Helsinki, Finland). Readings were carried out at daylight assisted by a standard light source. In the retrospective evaluation readings were subsequently carried out by three trained observers, of whom the first two performed the RDTs. The first two observers scored the test at 20 minutes, which is the recommended reading time, followed by the third observer. Photographs of the batches were taken immediately thereafter and within 25 minutes after application of the sample. The observers were blinded to the microscopy, PCR and each other’s results. In the prospective evaluation the laboratory technician who performed microscopy also performed the RDTs and was the single observer.

A scoring system was used to categorize line intensities: negative (N, no visible test line), faint (F, barely visible), weak (W, paler than the control line), medium (M, equal to the control line) and strong (S, stronger than the control line) [9]. Test results were based on consensus, *i.e.* an identical score by at least two out of three readers. In case of no consensus the photographs were reviewed to conclude.

### Data management and statistical analysis

Data was recorded on register forms and entered in a Microsoft Excel database (Microsoft Corporation, Redmond, Washington, USA). End points were sensitivity, specificity, inter-observer agreement and reproducibility. The interpretation of test results for *P. falciparum* and the non-*falciparum* species is shown in Tables 1 and 2. Sensitivity and specificity were calculated with 95% confidence interval (C.I.). Proportions were assessed for statistical significance using the two-tailed Fisher’s exact test and the McNemar test, for unpaired and paired panels respectively. A *p*-value < 0.05 was considered significant. To assess strength of associations between parasite densities, lot variation, duration of storage of the samples and the sensitivity specific per species, multivariate analysis was done with Stata 11.1 (StataCorp LP, Collage Station, USA). Inter-observer agreement for both results of positive and negative readings as well as for line intensity scorings was expressed by the percentage of overall agreement and by kappa values for each pair of observers. A kappa between 0.6 and 0.8 was considered a good agreement, higher than 0.8 was considered as excellent [9]. Test reproducibility was evaluated by testing 15 samples representing all species at varying parasite densities on six occasions.

### Additional analysis

All samples that generated invalid and unreadable results, all samples identified as species mismatch and all false-negative *P. falciparum* samples were tested again with CareStart pLDH in the same conditions as the initial testing.

### Package, labelling and instructions for use

Checklists for assessing quality of packaging, labelling and information insert were applied [10]. The Flesh Kincaid Grade Level was used to score the readability of the manufacturer’s instructions: it expresses the number of years of education that is needed to understand the text, based on measurement of length of words and sentences [10]. In addition, letter type (open versus closed), font size, and inter-line spacing were assessed as previously described [10].

### Ethical review

The study was approved by the Institutional Review Board of ITM and by the Ethical Committee of Antwerp University, Belgium.

## Results

### Retrospective evaluation

#### Sample collection

The retrospective evaluation consisted of 498 samples obtained in 498 patients with a median age of 37.0 years (range 5 months - 78 years), and a male to female ratio of 1.8:1. Seven children (1.4%) under the age of five were included. In 427 (85.7%) patients the travel history was known, 85.5% (365/427) of them had recently returned from sub-Saharan Africa and 9.8% (42/427) from Asia. Among the *P. falciparum* samples, there were 13 with pure gametocytaemia. The median parasite density of the remaining 234 *P. falciparum* samples was 869/μl (range 10–867,788/μl). The median (range) parasite densities for the 70 *P. vivax* samples, the 69 *P. ovale* and the 16 *P. malariae* were 701/μl (15–32,000/μl), 974/μl (10–10,000/μl) and 473/μl (0.1 - 6,096/μl) respectively.

### Test characteristics

No invalid test results were obtained. Two RDTs were scored as unreadable at first analysis, and the results obtained at repeat testing (which showed regular background clearing) were used. Table 3 shows the results of the test lines for all species. Of all *P. falciparum* samples nearly two-thirds (160/247, 64.8%) showed both Pf-pLDH and pan-pLDH test lines. Overall sensitivity for the detection of *P. falciparum* was 94.7% (Table 4). Sensitivity increased from 74.1% at parasite densities ≤ 100/μl to 98.1% and 98.7% at parasite densities > 100/μl and > 1,000/μl respectively (p < 0.001). The majority of false negative samples was observed among samples with pure gametocytaemia (2/12, 16.7%) and at parasite densities ≤ 100/μl (7/12, 58.3%). The remaining three false negative *P. falciparum* samples had parasite densities of 131/μl, 267/μl and 6,161/μl. When samples with pure gametocytaemia were excluded, overall sensitivity increased to 95.3%.

**Table 3:** Test results of CareStart pLDH for the retrospective evaluation (n = 498)

	**Pf-pLDH line positive number of samples (%)**		**Pf-pLDH line negative number of samples (%)**	
	**pan-pLDH line positive**	**pan-pLDH line negative**	**pan-pLDH line positive**	**pan-pLDH line negative**
*P. falciparum* (n = 247)	160 (64.8)	74 (30.0)	1 (0.4)*	12 (4.9)
*P. vivax* (n = 70)	9 (12.9)*		52 (74.3)	9 (12.9)
*P. ovale* (n = 69)			22 (31.9)	47 (68.1)
*P. malariae* (n = 16)			4 (25.0)	12 (75.0)
Negative (n = 96)				96 (100.0)

* Species mismatch

**Table 4:** **Accuracy of CareStart pLDH for the detection of*P. falciparum*, retrospective evaluation**

**Results of microscopy corrected by PCR**	**Number**	**Identified as *P. falciparum* by CareStart pLDH**	**% Sensitivity (95% C.I.)**	**% Specificity (95% C.I.)**
All *P. falciparum* samples	247	234	94.7 (91.2-97.2)	
Pure Gametocytemia	13	11	84.6 (54.6-98.1)	
Asexual parasite density 1-100/μl	27	20	74.1 (53.7-88.9)	
Asexual parasite density 101-200/μl	15	14	93.3 (68.1-99.8)	
Asexual parasite density 201–1,000/μl	34	33	97.1 (84.7-99.9)	
Asexual parasite density >1,000/μl	158	156	98.7 (95.5-99.9)	
Asexual parasite density >100/μl	207	203	98.1 (95.1-99.5)	
All other species and negative samples	251	9*		96.4 (93.3-98.4)

* All *P. vivax* samples.

For *P. vivax*, *P. ovale* and *P. malariae*, sensitivities were 74.3%, 31.9% and 25.0% respectively (Table 5). Sensitivities was higher at parasite densities above 500/μl compared to parasite densities below 500/μl. This difference reached statistical significance in the case of *P. ovale* (p = 0.001). Sensitivities at parasite densities above 500/μl remained below 50% for both *P. ovale* and *P. malariae*.

**Table 5:** **Accuray of CareStart pLDH for the detection of non-*falciparum*species, retrospective evaluation**

	**Number**	**Identified as non-*falciparum* species by CareStart pLDH**	**% Sensitivity (95% C.I.)**	**% Specificity (95% C.I.)**	
*P.vivax*	70	52	74.3 (62.4-84.0)		
Parasite density ≤ 500/μl	17	11	64.7 (38.3-85.8)		
Parasite density > 500/μl	53	41	77.4 (63.8-87.7)		
*P.ovale*	69	22	31.9 (21.2-44.2)		
Parasite density ≤ 500/μl	33	5	15.2 (5.1-31.9)		
Parasite density > 500/μl	36	17	47.2 (30.4-64.5)		
*P.malariae*	16	4	25.0 (7.3-52.4)		
Parasite density ≤ 500/μl	8	1	12.5 (0–52.7)		
Parasite density > 500/μl	8	3	37.5 (8.5-75.5)		
*P.falciparum* and negative samples	343	1		99.7 (98.4 -100)	
*P. falciparum*	247	1		99.6 (97.8 -100)	
No parasites detected	96	0		100 (96.2 -100)	

None of the 96 *Plasmodium* negative samples showed a test line (Tables 3 and 5). Wrong species identification (species mismatch) occurred in 10/402 (2.5%) samples: one *P. falciparum* sample (parasite density 2,043/μl) showed only the pan-pLDH test line and was consequently diagnosed as *Plasmodium* non-*falciparum*. Eight *P. vivax* samples (parasite densities > 1,000/μl) showed faint or weak Pf-pLDH lines in addition to the pan-pLDH test line, they were consequently diagnosed as *P. falciparum,* mixed infection not excluded. An additional *P. vivax* sample (568/μl) showed a Pf-pLDH line of strong line intensity in addition to the pan-pLDH line.

Multivariate analysis showed no impact of duration of storage of the samples on the diagnostic sensitivity. However, differences in RDT lot numbers were observed for *P. falciparum*; sensitivities were 96.5% and 86.7% for lot AIOIL and DIIML respectively (p = 0.019).

### Intensity of test lines

Among the *P. falciparum* samples, two-thirds (159/234, 67.9%) of visible test lines showed medium or strong Pf-pLDH test line intensities; faint test lines mostly (19/33, 57.6%) occurred at parasite densities < 100/μl (Additional file 1). Likewise, the presence of a unique Pf-pLDH line was mostly (62/74, 83.8%) observed in *P. falciparum* samples with parasite densities < 1,000/μl. The pan-pLDH test line displayed weaker intensities, with only 23.0% (37/161) of visible pan-pLDH test lines displaying medium or strong line intensities (Additional file 2).

For the non-*falciparum* samples that showed a visible pan-pLDH line (n = 87), the distribution of line intensities was as follows: faint 20.7%, weak 34.5%, medium 18.4% and strong 26.4%. Of note, *P. vivax* samples accounted for the vast majority (31/39, 79.4%) of pan-pLDH test lines with medium or strong line intensities ( Additional file 2).

### Inter-observer agreement and reproducibility

The overall agreement and kappa values between pairs of observers were excellent (> 0.80) for both positive and negative results and line intensity readings and for both Pf-pLDH and pan-pLDH test lines, except for one agreement that was scored as good (kappa value = 0.79). Test results were reproducible (Additional file 3 and Additional file 4) and all discordances in line intensity occurred within one category of difference.

### Prospective evaluation

#### Sample collection

The prospective evaluation consisted of 77 *Plasmodium* positive samples. Males represented 62.3% of the patients, and the median age was 35 years (range 4 months - 68 years). Three children (4%) under the age of five were included. In 51 patients (66.2%) the travel history was known, of which 76.5% (39/51) recently returned from Sub-Saharan Africa and 21.6% (11/51) from Asia. The sample collection contained all four *Plasmodium* species; among the *P. falciparum* samples (n = 49) there was one with pure gametocytaemia. The median parasite densities (range) of *P. falciparum, P. vivax* (n = 16)*, P. ovale* (n = 11) and *P. malariae* (n = 1) samples were 6,687/μl (27 – 665,432/μl), 2,473/μl (507–20,953/μl), 858/μl (22–7,292/μl) and 612/μl respectively.

### Test characteristics

There were no invalid neither unreadable test results. Table 6 shows the number of visible test lines for all species. Of all *P. falciparum* samples two-thirds (33/49, 67.3%) displayed both Pf-pLDH and pan-pLDH test lines.

**Table 6:** Test results of CareStart pLDH for the prospective evaluation (n = 77)

	**Pf-pLDH line positive number of samples (%)**		**Pf-pLDH line negative number of samples (%)**	
	**pan-pLDH line positive**	**pan-pLDH line negative**	**pan-pLDH line positive**	**pan-pLDH line negative**
*P. falciparum* (n = 49)	33 (67.4)	13 (26.5)		3 (6.1)
*P. vivax* (n = 16)	2 (12.5)*		14 (87.5)	
*P. ovale* (n = 11)			7 (63.6)	4 (36.4)
*P. malariae* (n = 1)			1 (100.0)	

* Species mismatch.

Table 7 displays the sensitivity for the *P. falciparum* and the non-*falciparum* samples. Seven false negative results occurred, of which three *P. falciparum* samples with parasite densities of 32 μ/l, 130 μ/l and 267 μ/l and four *P. ovale* samples with parasite densities of 22 μ/l, 278 μ/l, 609 μ/l and 1,516 μ/. Two *P. vivax* samples (2/77, 2.6%, with parasite densities of 2,508/μl and 11,578/μl) showed a weak and a medium Pf-pLDH test line respectively in addition to a pan-pLDH line. Both samples were obtained from members of a family who had recently returned from India.

**Table 7:** **Sensitivity of CareStart pLDH for the detection of*Plasmodium*, prospective evaluation**

		**Number**	**Correctly identified by CareStart pLDH**	**% Sensitivity (95% C.I.)**
*All P. falciparum* samples		49	46	91.8 (80.4-97.7)
Pure gametocytemia		1	1	
Asexual parasite density 1-100/μl		6	5	
Asexual parasite density 101-200/μl		1	0	
Asexual parasite density 201–1,000/μl		5	4	
Asexual parasite density >1,000/μl		36	36	100 (90.3-100)
Asexual parasite density >100/μl		42	40	95.2 (83.8-99.4)
All non-falciparum species		28	22*	78.6 (59.1-91.7)
Parasite density ≤ 500/μl		3	1	
Parasite density > 500/μl		25	21	84.0 (63.9-95.5)

* Miss diagnosed two *P.vivax* samples that showed both test lines, and missed four *P. ovale* samples.

In Table 8 the diagnostic sensitivities of the CareStart pLDH, OptiMAL and SDFK60 are presented. For *P. falciparum*, diagnostic sensitivity of CareStart pLDH was higher as compared to OptiMAL (p = 0.008) and tended to be lower compared to SDFK60 (p = 0.08). OptiMAL and SDFK60 both detected all 16 *P. vivax* samples, whereas CareStart pLDH missed two of them (p = 0.2). Conversely, CareStart pLDH performed significantly better for the detection of *P. ovale* compared to OptiMAL (p = 0.03) and SDFK60 (p = 0.01).

**Table 8:** Diagnostic sensitivities of the different RDTs for each species, prospective evaluation (n = 76)*

	**RDT brand**	**Number correctly identified by RDT**	**% Sensitivity (95% C.I.)**		
*P. falciparum (n = 49)*	CareStart pLDH	46	93.9 (83.1-98.7)		
					
	OptiMAL	39	79.6 (65.7-98.8)		
	SDFK60	49	100 (92.8-100)		
*P. vivax (n = 16)*	CareStart pLDH	14	87.5 (61.7-98.5)		
	OptiMAL	16	100 (79.4-100)		
	SDFK60	16	100 (79.4-100)		
*P. ovale (n = 10)*	CareStart pLDH	6	60.0 (26.2-87.8)		
					
	OptiMAL	1	10.0 (0–44.5)		
	SDFK60	0	0 (0–30.9)		
*P. malariae (n = 1)*	CareStart pLDH	1			
	OptiMAL	1			
	SDFK60	1			

* One *P. ovale* sample was not tested with OptiMAL and SDFK60.

### Additional analysis

Eleven out of 12 false negative *P. falciparum* samples were available for retesting: two of them (parasite densities 32/μl and 6,161/μl) showed a faint or weak Pf-pLDH test line, the other nine samples (all with parasite densities below 1,000/μl) showed no visible test line, as upon initial testing.

Retesting of samples with species mismatch revealed the following: the *P. falciparum* sample initially identified as non-*falciparum* (parasite density 2,043/μl) consistently showed a unique pan-pLDH test line upon repeat testing. Among the *P. vivax* samples that showed both Pf-pLDH and pan-pLDH test lines upon initial testing (n = 9), two yielded identical results while seven did not show the Pf-pLDH line upon retesting. Of note, six of the latter ones were tested with lot number DIIML whereas initial testing was performed with lot number AIOIL.

### Package, labelling and information insert

With regard to quality of packaging, labelling and information inserts, the following observations were made: a CE representative was not mentioned and the CE mark was not printed in the correct format. Secondly, the names of the test kit as displayed on information inserts, boxes, the device blisters and buffer vials showed differences (box: “pLDH 3 lines (pan/Pf)”; package insert: “CareStart pLDH”, device blister: “pLDH (pan/Pf) ”, device: no name; buffer “buffer assay”). Further, box and labels were not humidity resistant and the label on the buffer vials did not list the test kits name, expiry date nor lot number.

**Figure 1:**
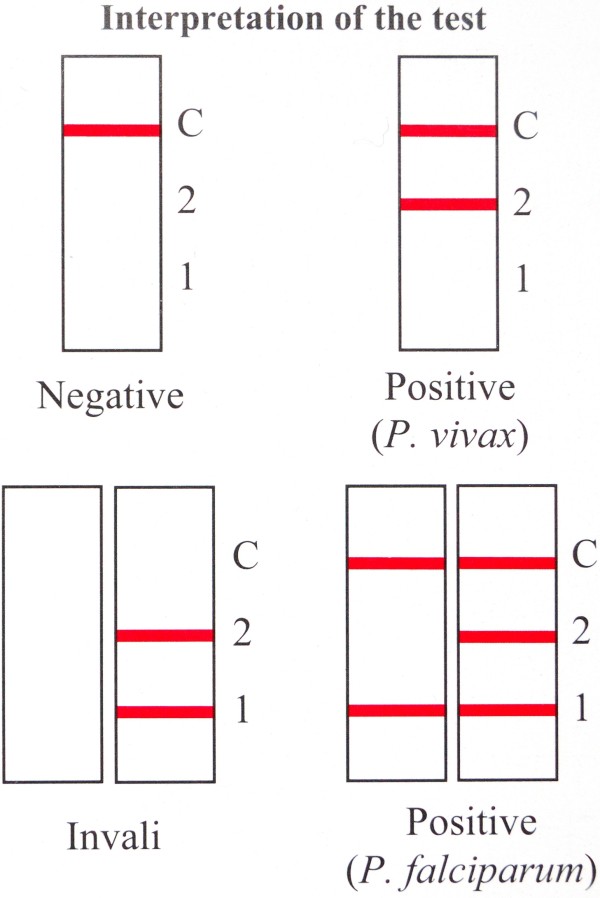
**Information insert of CareStart pLDH, instructions for use, interpretation: the figure displays the interpretation of test results.** The following shortcomings are observed: (i) upper right: C + 2: correct interpretation is “*Plasmodium* non-*falciparum*” instead of “*P. vivax*”; (ii) under right: C + 2 + 1: correct interpretation is “*P. falciparum*, mixed species infection is not excluded”. Not all line combinations pointing to an invalid test are depicted and there is a typographical error in the term “invalid” (under left).

The instructions for use did not mention (i) to check expiry date and saturation of silica gel, (ii) to write the patient identification on the cassette and (iii) to use only the buffer vial provided in the kit. In addition, the illustrations of the depicted cassettes showed slight differences with the real RDT device: (i) the labelling of wells and reading windows was not depicted and (ii) the shape of the sample well was depicted as oval instead of rectangular. The test lines were depicted in a bright red color, whereas in reality they are purple-red. In addition, there were shortcomings in the interpretation of test lines (Figure 1). Readability expressed as Flesh Kincaid Grade Level was 8.68, a closed letter type was used and font size was 8 with an interline spacing of 2.

## Discussion

The present study assessed the performance of CareStart pLDH in a reference setting, retrospectively on stored and prospectively on fresh samples, obtained in international travelers suspected of malaria. Overall sensitivity for *P. falciparum* was > 90%, and reached > 98% at parasite densities above 100/μl. False-negative results mainly occurred at parasite densities < 100/μl. One *P. falciparum* sample was wrongly diagnosed as a non-*falciparum* species. Overall sensitivity for *P. vivax* was good, but poor for *P. ovale* and *P. malariae*.

Evaluating a RDT in a reference setting is a logic step preceding field studies though it has inherent limitations [9,11-13]. For instance, the retrospective design made it impossible to retrieve clinical information such as treatment and interfering factors like rheumatoid factor that might explain for unexpected results. Next, unlike HRP-2 - which is a very stable antigen [14] - pLDH may degrade during long storage [12], although such an effect was not demonstrated in the present study. Finally, the application of strict interpretation criteria influenced test outcomes: first, *P. falciparum* samples with pure gametocytaemia were included among the positive samples. This is meaningful in the scope of travel medicine [15], but tends to decrease the diagnostic sensitivity of the RDT studied as was the case in the present study. Second, species mismatches were considered as false negatives, despite the fact that the diagnosis of malaria was confirmed. Categorizing the samples with species mismatch as true positives would have increased the sensitivity for *P. vivax* in the retrospective evaluation from 74.3% to 87.1%.

CareStart pLDH was previously evaluated by the World Health Organization (WHO) and the Foundation for Innovative New Diagnostics (FIND) [16]: detection of *P. falciparum* and *P. vivax* was assessed with diluted samples at fixed parasite densities. The detection rates at low parasite densities (200/μl) were 88.9% and 91.4% for *P. falciparum* and *P. vivax* respectively. At high parasite densities (2,000/μl or 5,000/μl) the detection rate was 100% for both species. In addition, three field studies have reported on CareStart pLDH: in Myanmar, Ashley and coworkers reported sensitivities of 90.5% and 78.9% for *P. falciparum* and *P. vivax* samples respectively [17]. In Madagascar, Ratsimbasoa and coworkers found sensitivities of 97.0% for *P. falciparum* but included too few non-*falciparum* samples for calculation of sensitivity [18]. The high sensitivity for *P. falciparum* in their study as compared to the present can be explained by several facts: in the study of Ratsimbasoa, (i) samples with pure gametocytaemia were excluded, (ii) any visible test line was considered as a correct identification, and (iii) the mean parasite density (6,564/μl) was higher [18]. A third study from Sierra Leone that examined children under five years of age: CareStart pLDH showed a sensitivity of 99.4% for *P. falciparum*[19]. High parasite densities (median 264,000/μl) in that study may have accounted for the high sensitivity.

Unlike these previous studies the present study included all four *Plasmodium* species.

CareStart pLDH tended to a lower sensitivity for *P. falciparum* when run side-to-side with the HRP-2 based SDFK60. This is not unexpected, as HRP-2 based RDTs are ascribed a higher sensitivity at low parasite densities [20]. For *P. falciparum*, all but one false-negative results with CareStart pLDH occurred in samples with parasite densities below 100/μl. Sensitivities of CareStart pLDH for the detection of *P. vivax* were lower in the retrospective as compared to the prospective evaluation. Apparently, this was not due to an effect of samples storage at – 70°C, but could be explained by (i) lower parasite densities in the retrospective panel and (ii) cross-reaction of *P. vivax* samples with the Pf-pLDH line (tested with lot AIOIL), which were consequently categorized as false-negatives in the retrospective evaluation. If these cross-reactions were disregarded, the sensitivity of CareStart pLDH for the detection of *P. vivax* was good. Despite the fact that most cross-reactions with the Pf-pLDH test line occurred in lot AIOIL, they were also observed with lot DIIML and both observations – lot-to-lot differences and cross reactions – are of concern. Sensitivity for the *P. ovale* and *P. malariae* species was in line with results described for other RDTs [12,15,21,22]. Of note, in the prospective evaluation, CareStart pLDH performed better than OptiMAL and SDFK60 for the detection of *P. ovale*. The different options proposed recently by Piper and co-authors for the improvement of the specificity and the sensitivity of the pLDH based malaria RDTs (optimization of the buffer conditions and solid support matrices, or even the use of alternative antibodies that have different binding characteristics) should be taken into account by the manufacturer [23].

The differences of sensitivity for the *Plasmodium* species were reflected in the distribution of line intensities: for *P. falciparum* samples, approximately two-thirds of Pf-pLDH lines were well visible (strong to medium line intensities), as were half of pan-pLDH lines in the case of *P. vivax*. By contrast, the majority of *P. ovale* and *P. malariae* samples with visible pan-pLDH lines had faint or weak line intensities. Faint or weak test lines are a concern particularly in resource limited settings, as they are difficult to be discerned in poor light conditions and tend to be disregarded as negative. The lower sensitivity of pan-pLDH test line intensities in the case of *P. ovale* and *P. malariae* compared to *P. vivax* may be caused by a lower affinity of the pan-pLDH antibodies for the former species [23].

In addition to the differences in cross-reactions of *P. vivax* with the Pf-pLDH line mentioned above, there was also a difference in diagnostic sensitivity for detection of *P. falciparum* between both lots tested. Lot-to-lot variation in RDTs is a well-known issue in performance, monitoring and quality control of RDTs [13,16]. As slight – but important – differences between lots such as those presently observed will probably go undetected by routine lot control procedures, efforts should be made at the level of manufacturing and post-marketing surveillance to assure equal performance of different RDT lots.

Improvements in package and labelling of CareStart pLDH should be considered, which can be done even at low-cost. The similarity of RDT boxes of different CareStart RDTs from AccessBio was confusing, especially since the individual test names were not printed on the RDT blisters, cassettes and buffer vials. The interpretation section of the package insert contained the same error as previously described for the CareStart Malaria pLDH/HRP2 kit [4]. As was the case for other instruction leaflets described previously, the CareStart pLDH letter type (closed), font size (8) and Flesh Kincaid score (8.9) were below expectations, particularly when use in resource poor settings is intended: as a comparison, for patient education materials and health related information sheets, font sizes of ≥ 12 and Flesh Kincaid Grade Levels ≤ 6 are recommended [10].

What can be the place of CareStart pLDH in the diagnostic setting? Most RDTs that diagnose *P. falciparum* are targeting HRP-2, which is known to be detected at lower parasite densities compared to Pf-pLDH [20]. One comparative study described lower heat stability for the Pf-pLDH based RDTs compared to HRP-2 based tests [24], but the recent Round 3 of the WHO/FIND evaluation did not confirm this association and CareStart pLDH scored equal to HRP-2 based RDTs in the heat-stability assessment [16]. HRP-2 based RDTs have their limitations: HRP-2 gene deletions described in the Peruvian Amazon may impede their use [25], persistence of HRP-2 up to 43 days after a successful treatment decreases the diagnostic value of a positive result in endemic settings [20] and unlike Pf-pLDH based RDTs they are susceptible to the prozone effect (false negative or faint test lines at high parasite densities) [26,27]. In combination with its good performance for the detection of *P. vivax*, CareStart pLDH may be an alternative to other well-described HRP-2 three band RDTs in the diagnostic setting in non-endemic areas. Despite being better than the other two RDTs which were run side-to-side, it should be reminded that its performance for the detection of *P. ovale* and *P. malariae* is poor.

## Abbreviations

EDTA, Ethylene diamine tetra-acetic acid; FIND, Foundation for Innovative New Diagnostics; HRP-2, Histidine-rich protein 2; ISO, International organization for standardization; ITM, Institute of Tropical Medicine; P, Plasmodium; Pan-pLDH, Pan Plasmodium-specific parasite lactate dehydrogenase; PCR, Polymerase chain reaction; Pf-pLDH, Plasmodium falciparum-specific parasite lactate dehydrogenase; pLDH, Parasite lactate dehydrogenase; Pv-pLDH, Plasmodium vivax-specific parasite lactate dehydrogenase; RDT(s), Rapid diagnostic test(s); STARD, Standards for the reporting of diagnostic accuracy studies; WHO, World Health Organization.

## Competing interests

The authors declare that they have no competing interests.

## Authors’ contributions

PG and JJ designed the study protocol, MVE and EB organized prospective sample collection. MH, JM, AS and PG carried out the RDT test evaluations, LC performed the PCR analysis, MH, PG, JM and JJ analyzed and interpreted the results. MH and JJ drafted the manuscript. All authors critically reviewed the manuscript and approved the final manuscript.

## Supplementary Material

Additional file 1 Line intensity consensus for the CareStart pLDH for Pf-pLDH, retrospective evaluation. Click here for file

Additional file 2 Line intensity consensus for the CareStart pLDH for pan-pLDH, retrospective evaluation. Click here for file

Additional file 3 Reproducibility of the CareStart pLDH: Pf-pLDH line intensities, retrospective evaluation. Click here for file

Additional file 4 Reproducibility of the CareStart pLDH: pan-pLDH line intensities, retrospective evaluation. Click here for file
